# Systematic review and meta-analysis of video-assisted thoracoscopic surgery segmentectomy versus lobectomy for stage I non–small cell lung cancer

**DOI:** 10.1186/s12957-020-01814-x

**Published:** 2020-02-27

**Authors:** Weibiao Zeng, Wenxiong Zhang, Jianyong Zhang, Guangmiao You, Yu’ang Mao, Jianjun Xu, Dongliang Yu, Jinhua Peng, Yiping Wei

**Affiliations:** 1 Department of Cardiothoracic Surgery, The Second Affiliated Hospital of Nanchang University, Nanchang, China; 2 Department of General Surgery, The Affiliated Hospital of Guizhou Medical University, Guiyang, Guizhou Province China

**Keywords:** Lung cancer, NSCLC, VATS segmentectomy, Meta-analysis

## Abstract

**Background:**

Whether video-assisted thoracoscopic surgery (VATS) segmentectomy and VATS lobectomy provide similar perioperative and oncological outcomes in stage I non–small cell lung cancer (NSCLC) is still controversial.

**Methods:**

Meta-analysis of 12 studies comparing outcomes after VATS lobectomy and VATS segmentectomy for stage I NSCLC. Data were analyzed by the RevMan 5.3 software.

**Results:**

Disease-free survival (HR 1.19, 95% CI 0.89 to 1.33, *P* = 0.39), overall survival (HR 1.11, 95% CI 0.89 to 1.38, *P* = 0.36), postoperative complications (OR = 1.10, 95% CI 0.69 to 1.75, *P* = 0.7), intraoperative blood loss (MD = 3.87, 95% CI − 10.21 to 17.94, *P* = 0.59), operative time (MD = 10.89, 95% CI − 13.04 to 34.82, *P* = 0.37), air leak > 5 days (OR = 1.20, 95% CI 0.66 to 2.17, *P* = 0.55), and in-hospital mortality (OR = 1.67, 95% CI 0.39 to 7.16, *P* = 0.49) were comparable between the groups. Postoperative hospital stay (MD = − 0.69, 95% CI − 1.19 to − 0.19, *P* = 0.007) and number of dissected lymph nodes (MD = − 6.44, 95%CI − 9.49 to − 3.40, *P* < 0.0001) were significantly lower in VATS segmentectomy patients.

**Conclusions:**

VATS segmentectomy and VATS lobectomy provide similar oncological and perioperative outcomes for stage I NSCLC patients.

This systematic review was registered on PROSPERO and can be accessed at http://www.crd.york.ac.uk/PROSPERO/display_record.php?ID = CRD42019133398.

## Background

Advances in screening techniques have led to a marked increase in the number of small peripheral lung lesions being detected [1]. About 10% of these lesions turn out to be non–small cell lung cancers (NSCLC). Lobectomy with radical lymph node dissection has been the preferred management for stage I NSCLC since 1995, when the North American Lung Cancer Study Group [2] reported better survival with lobectomy than with sublobectomy. The authors of the study recommended sublobectomy only for patients with limited cardiopulmonary reserve. However, the study included patients with various clinical stages and did not discriminate sublobectomy from wedge resection and segmentectomy, and so the conclusions have been questioned by some experts. Recently, there has been a revival of interest in sublobectomy, and segmentectomy in particular, for management of stage I NSCLC. Segmentectomy preserves lung tissue and so obviously protects lung function [3, 4]; in addition, video-assisted thoracoscopic surgery (VATS) segmentectomy [5–8], which is the preferred procedure, causes less postoperative pain and requires shorter hospitalization than thoracotomy. However, it remains unclear whether perioperative safety and long-term survival are comparable between stage I NSCLC patients treated with VATS segmentectomy and VATS lobectomy. Therefore, we performed this meta-analysis to determine whether perioperative outcomes (such as postoperative complications, intraoperative blood loss, air leak) and survival (disease-free survival [DFS] and overall survival [OS]) were similar in stage I NSCLC patients treated with VATS segmentectomy and VATS lobectomy.

## Materials and methods

### Search strategy

Two investigators independently searched PubMed, Web of Science, ScienceDirect, The Cochrane Library, Scopus, and Google Scholar to identify relevant papers published between January 1990 and April 2019. The following keywords were used: “lobectomy AND segmentectomy” “lung cancer OR lung neoplasm OR non–small cell lung cancer OR NSCLC”, and “video-assisted thoracic surgery OR VATS”. There were no limits placed on study design or publication status (published or unpublished). The search strategy is comprehensively described in Additional file 1.

### Selection criteria

Studies were eligible for inclusion in this meta-analysis if they (1) were in English, (2) only included patients with clinical stage I NSCLC, and (3) compared the perioperative and/or survival outcomes (follow-up time ≥ 5 years) of patients treated with VATS segmentectomy and VATS lobectomy. When the same data or data subsets were reported in more than one study, the one with the most details or the one most recently published was chosen. Case-only designs, case reports, systematic reviews, meta-analyses, and animal studies were excluded.

### Data extraction

Two investigators independently went through each eligible study and recorded data on the following: name of first author, year of publication, geographic area, study design, DFS, OS, postoperative complications, intraoperative blood loss, operation time, postoperative hospital stay, air leak (> 5 days), in-hospital mortality, and number of lymph nodes dissected.

### Quality assessment for included studies

The quality of each study was graded independently by two investigators using the Newcastle–Ottawa Scale (NOS, for nonrandomized studies). The NOS analyzes three items—selection, comparability, and exposure—to evaluate study quality. The maximum possible score is 4 for selection, 2 for comparability, and 3 for exposure. A total score of 8 or 9 indicates high quality, and a score of 6 or 7 indicates medium quality.

Details of the protocol for this systematic review were registered on PROSPERO and can be accessed at http://www.crd.york.ac.uk/PROSPERO/display_record.php?ID = CRD42019133398. This study is presented in accordance with the Preferred Reporting Items for Systematic Reviews and Meta-Analyses (PRISMA) Statement.

### Statistical analysis

Statistical analysis was performed using Review Manager 5.3 (The Nordic Cochrane Centre, The Cochrane Collaboration, Copenhagen, Denmark) and SPSS 18.0 (SPSS Inc., Chicago, IL, USA). Survival data (OS and DFS) were analyzed by using the hazard ratio (HR) and its standard error (SE). If the HR data were unable to be extracted directly from the included studies, we extracted data from Kaplan–Meier curves and calculated the data according to method provided by Tierney et al. [9]. The Kaplan–Meier curves were read by Engauge Digitizer version 4.1 (software downloaded from http://sourceforge.net/projects/digitizer/files/Engauge%20Digitizer/digitizer-4.1/). All calculations were performed independently by two of the authors; disagreements were settled by discussion. Higgins *I*^2^ statistic was used to evaluate heterogeneity among the included studies. If no significant heterogeneity was detected (*I*^2^ < 50%, *P* > 0.1), the fixed-effects model was used to pool studies; otherwise, the random-effect model was used. For some of the studies, the original data were recalculated. Funnel plots were used to assess publication bias.

## Result

### Included studies

**Fig. 1:**
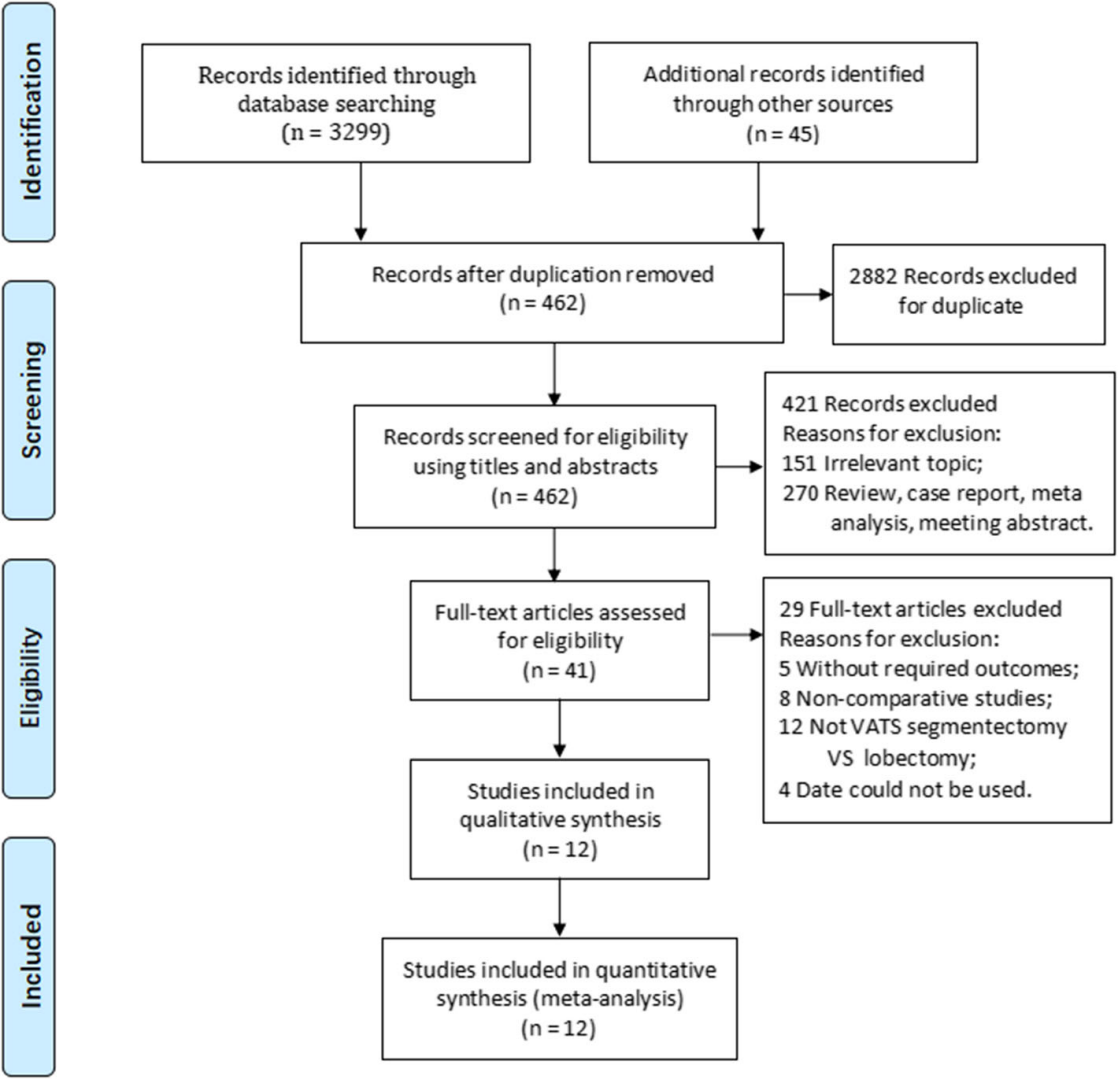
Flow diagram showing the process of selection of studies

A total of 3299 publications were identified with the electronic search of the databases and the manual search of reference lists. Of these, 12 articles met our eligibility criteria (Fig. 1). These 12 articles involved a total of 2313 patients, with 750 who underwent VATS segmentectomy and 1563 who underwent VATS lobectomy. All 12 articles were retrospective studies. By the Newcastle–Ottawa Scale, six articles were graded as good quality and six as medium quality (details are presented in Additional file 2). Table 1 presents the characteristics of the 12 articles.

**Table 1:** Summary of the 12 studies included in the meta-analysis

First author	Study type	Region	Research period	Clinical stage	Staging procedures	Surgery		Outcomes reported	Quality score
						VATS segmentectomy	VATS lobectomy		
Hwang [10]	Retrospective	Korea	2015	AIS IA IB	CT	94	94	(1) (2) (3) (5) (6) (8)(9)	9
Echavarria [11]	Retrospective	USA	2016	I	Unknown	43	208	(3) (4) (5) (6) (7) (8)	8
Landreneau [12]	Retrospective	Australia	2014	IA IB	Unknown	170	170	(1) (2)	8
Nakamura [13]	Retrospective	Japan	2011	I	Unknown	38	289	(2)	7
Roman [14]	Retrospective	UK	2019	IA IB	All CT and PET	40	44	(2)	8
Shapiro [15]	Retrospective	USA	2009	IA IB	All CT and partial PET	31	113	(1) (2) (3) (6) (7) (8)	7
Song [16]	Retrospective	Japan	2018	IA	All CT and 87.7% PET	41	41	(1) (3) (4) (5) (6) (7) (8) (9)	9
Soukiasian [17]	Retrospective	USA	2012	IA IB	Unknown	73	266	(2) (3) (7)	7
Tsubokawa [18]	Retrospective	Japan	2018	IA IB	All CT and PET	52	44	(1) (2) (3) (4) (9)	7
Wang [19]	Retrospective	China	2013	IA IB	All CT and PET	5	14	(4) (6)	7
Yamashita [20]	Retrospective	Japan	2012	IA	CT	90	124	(1) (2) (4) (5) (8) (9)	8
Zhong [21]	Retrospective	China	2012	IA	CT and partial PET	39	81	(1) (2) (3) (4) (5)(6) (7) (8) (9)	7

*Abbreviations*: *(1)* Disease-free survival, *(2)* overall survival, *(3)* postoperative complications, *(4)* perioperative blood loss, *(5)* operation time, *(6)* hospital stay, *(7)* air leak (> 5 days), *(8)* in-hospital mortality, *(9)* number of dissected lymph nodes, *VATS* video-assisted thoracoscopic surgery

### Primary outcome measures

#### Disease-free survival

**Fig. 2:**
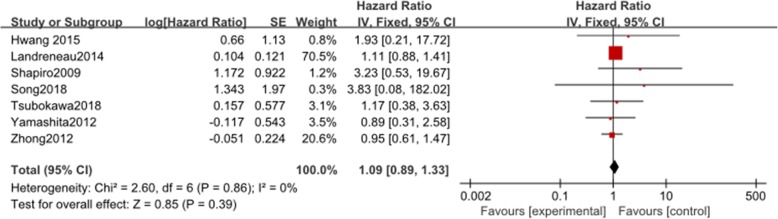
Forest plot for disease-free survival of the VATS segmentectomy and VATS lobectomy groups in the studies analyzed

Seven studies reported data on DFS. These 7 studies involved a total of 1184 patients, among whom 517 received VATS segmentectomy and 667 patients received VATS lobectomy. There was no heterogeneity among the studies (*I*^2^ = 0%, *P* = 0.86). The combined HR for DFS was 1.09 (95% CI 0.89 to 1.33). DFS was not significantly different between the two groups (*P* = 0.39, Fig. 2).

#### Overall survival

**Fig. 3:**
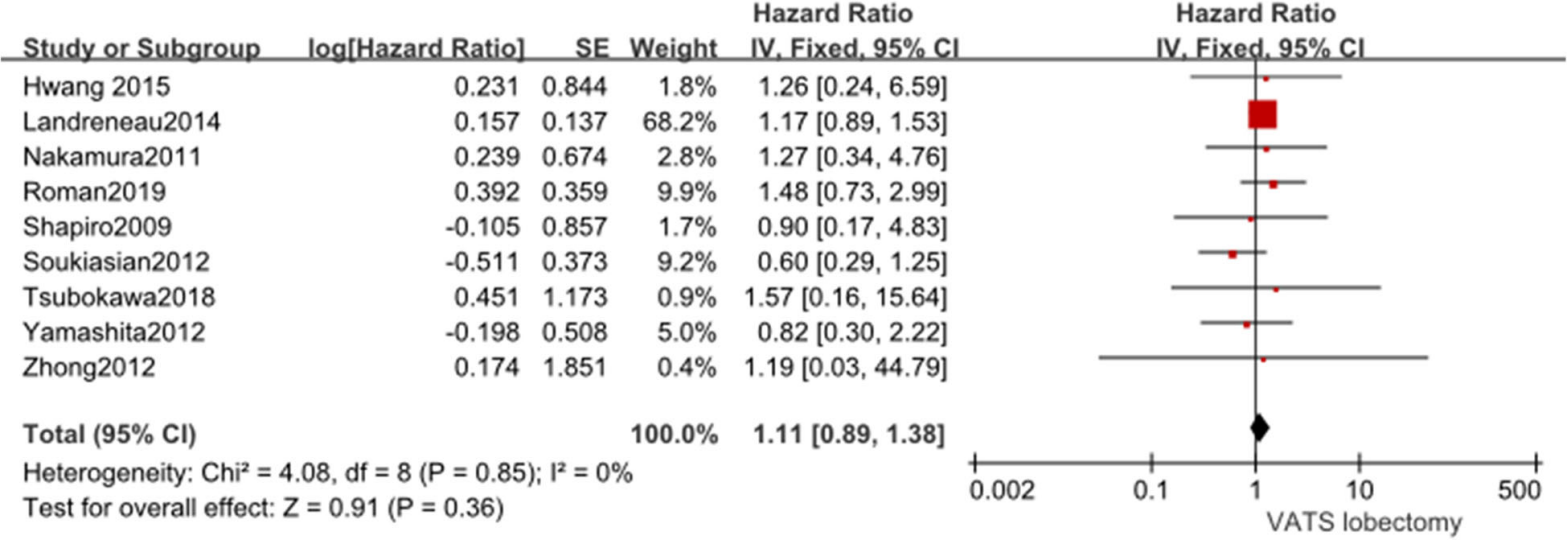
Forest plot for overall survival of the VATS segmentectomy and VATS lobectomy groups in the studies analyzed

Nine studies reported data on OS. These 9 studies involved a total of 2160 patients, among whom 935 received VATS segmentectomy and 1225 received VATS lobectomy. There was no heterogeneity among the studies (*I*^2^ = 0%, *P* = 0.85). The combined HR for OS was 1.11 (95% CI 0.89 to 1.38). OS was not significantly different between the two groups (*P* = 0.36, Fig. 3).

#### Postoperative complications

**Fig. 4:**
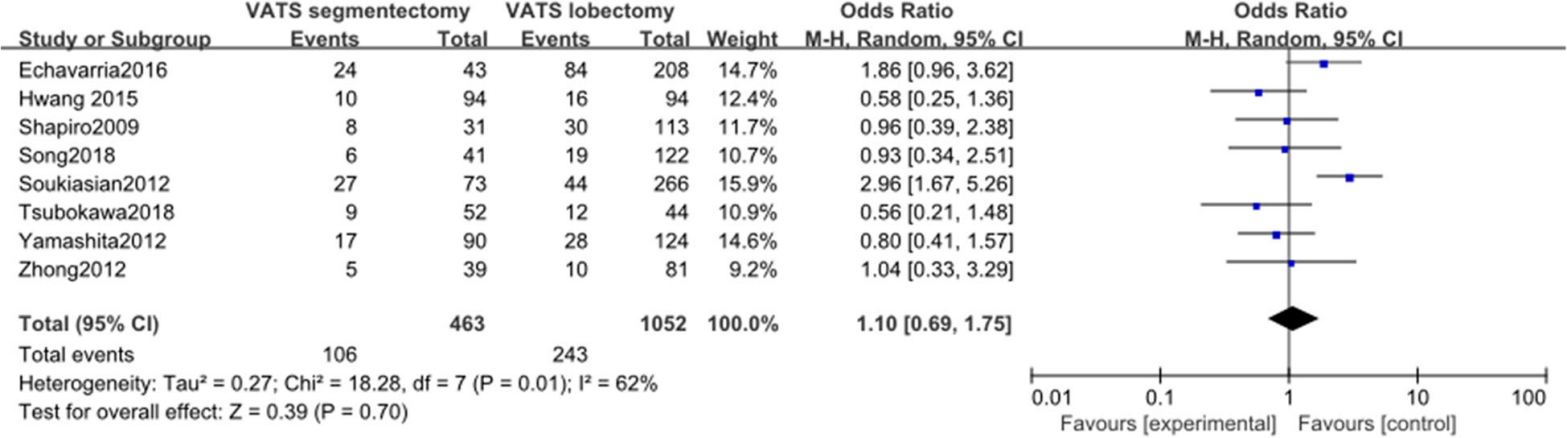
Forest plot for postoperative complications of the VATS segmentectomy and VATS lobectomy groups in the studies analyzed

Eight articles reported data on postoperative complications. These 8 studies included a total of 1515 patients, among whom 463 received VATS segmentectomy and 1052 received VATS lobectomy. There was significant heterogeneity among the studies (*I*^2^ = 62%, *P* = 0.01). The incidence of postoperative complications was not significantly different between the two groups (OR = 1.10, 95% CI 0.69 to 1.75, *P* = 0.70, Fig. 4).

#### Postoperative hospital stay

**Fig. 5:**
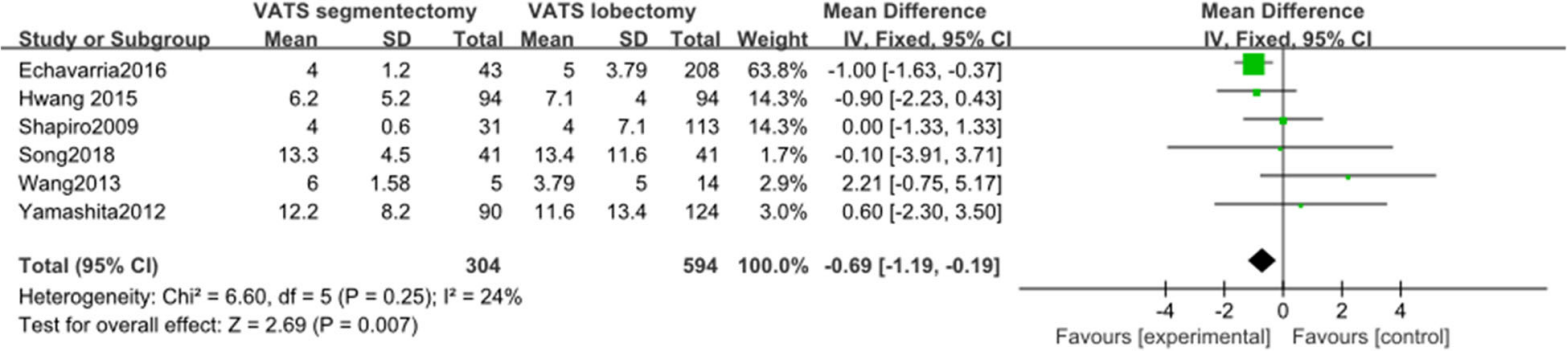
Forest plot for postoperative hospital stay of the VATS segmentectomy and VATS lobectomy groups in the studies analyzed

Six articles reported data on postoperative hospital stay. These 6 studies involved a total of 898 patients, among whom 304 received VATS segmentectomy and 594 received VATS lobectomy. There was no significant heterogeneity among the studies (*I*^2^ = 24%, *P* = 0.25). Postoperative hospital stay was shorter in VATS segmentectomy patients than in VATS lobectomy patients. The difference in postoperative hospital stay between the two groups was statistically significant (MD = − 6.44, 95% CI − 9.49 to − 3.40, *P* = 0.007, Fig. 5).

#### Intraoperative blood loss

**Fig. 6:**
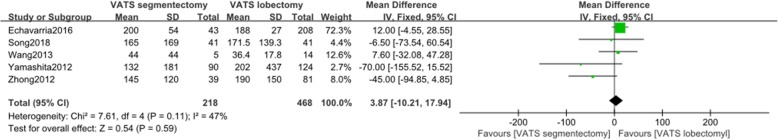
Forest plot for intraoperative blood loss of the VATS segmentectomy and VATS lobectomy groups in the studies analyzed

Five articles reported data on intraoperative blood loss. These 5 studies involved a total of 686 patients, among whom 218 received VATS segmentectomy and 468 received VATS lobectomy. There was no significant heterogeneity between the studies (*I*^2^ = 47%, *P* = 0.11). The mean difference in intraoperative blood loss between the two groups was not statistically significant (MD = 3.87, 95% CI − 10.21 to 17.94, *P* = 0.59, Fig. 6).

#### Operation time

**Fig. 7:**
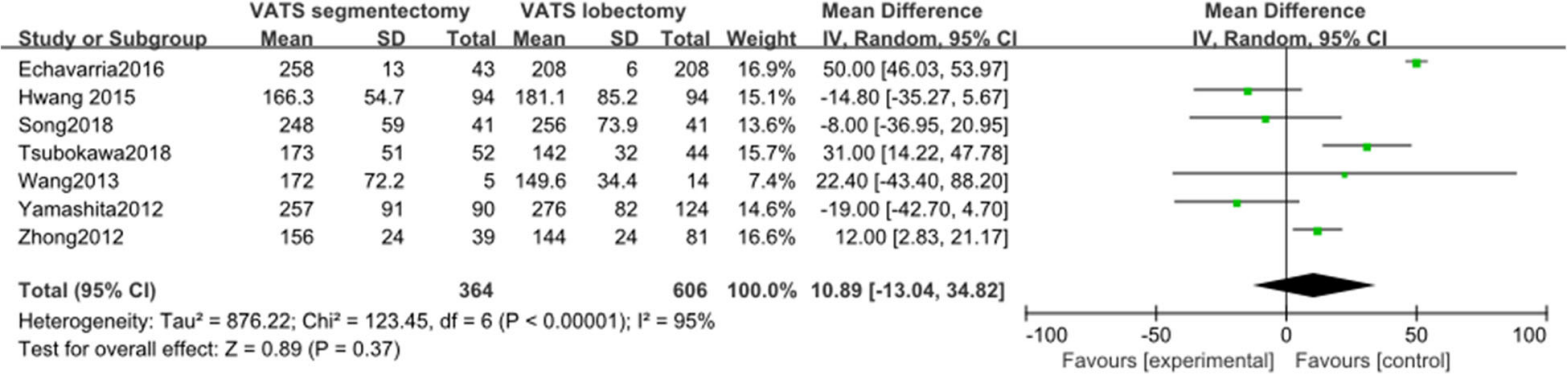
Forest plot for operation time of the VATS segmentectomy and VATS lobectomy groups in the studies analyzed

Seven articles reported data on operation time. These 7 studies involved a total of 970 patients, among whom 364 received VATS segmentectomy and 606 received VATS lobectomy. There was significant heterogeneity among the studies (*I*^2^ = 95%, *P* < 0.001). The mean difference in operation time between the two groups was not statistically significant (MD = 10.89, 95% CI − 13.04 to 34.82, *P* = 0.37, Fig. 7)

#### Air leak (> 5 days)

**Fig. 8:**
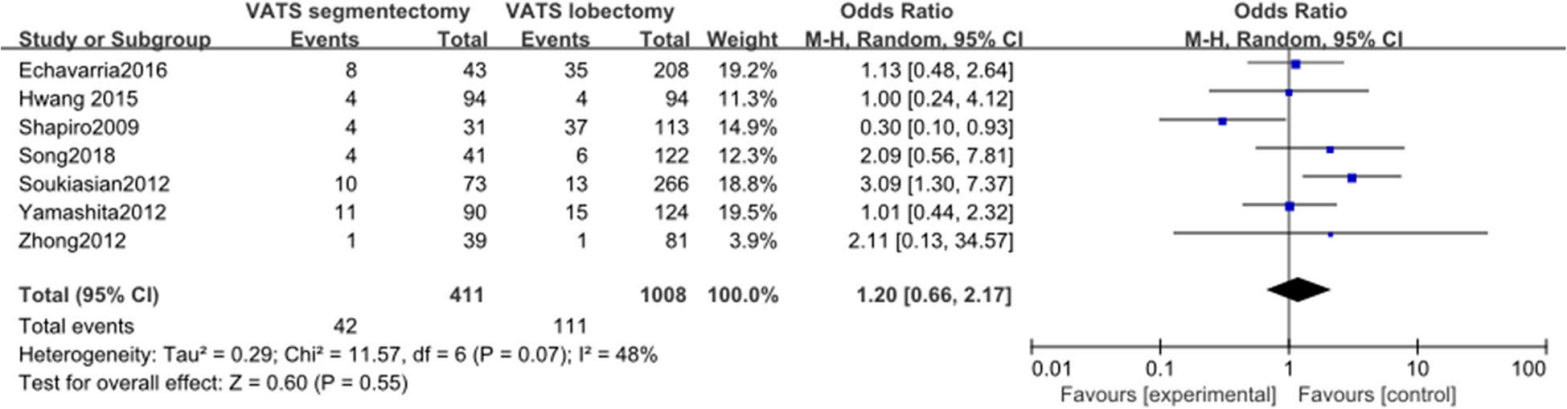
Forest plot for air leak (> 5 days) of the VATS segmentectomy and VATS lobectomy groups in the studies analyzed

Seven articles reported data on air leak. These 7 studies involved a total of 1419 patients, among whom 411 received VATS segmentectomy and 1008 received VATS lobectomy. There was no significant heterogeneity among the studies (*I*^2^ = 48%, *P* = 0.07). The difference in air leak between the two groups was not statistically significant (OR = 1.20, 95% CI 0.66 to 2.17, *P* = 0.55, Fig. 8).

#### In-hospital mortality

**Fig. 9:**
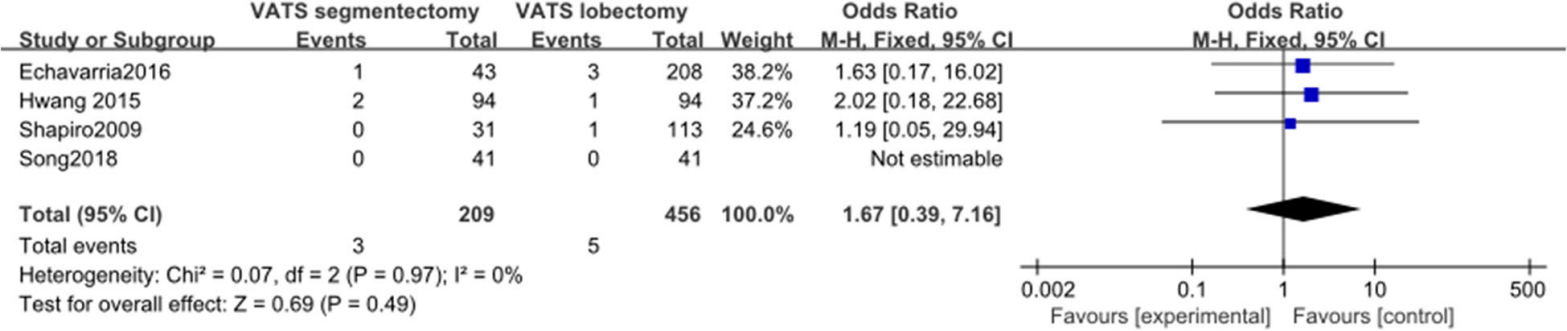
Forest plot for in-hospital mortality of the VATS segmentectomy and VATS lobectomy groups in the studies analyzed

Four articles were reported data on in-hospital mortality. These 4 studies involved a total of 665 patients, among whom 209 received VATS segmentectomy and 456 received VATS lobectomy. There was no heterogeneity among the studies (*I*^2^ = 0%, *P* = 0.97). The difference in incidence of air leak between the two groups was not statistically significant (OR = 1.67, 95% CI 0.39 to 7.16, *P* = 0.49, Fig. 9).

#### Number of lymph nodes dissected

**Fig. 10:**
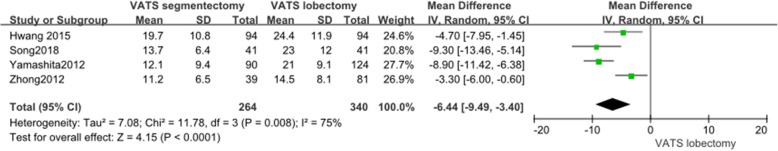
Forest plot for dissected lymph nodes of VATS segmentectomy and VATS lobectomy groups in the studies analyzed

Four articles reported data on dissected lymph nodes. These 4 studies involved a total of 604 patients, among whom 264 received VATS segmentectomy and 340 received VATS lobectomy. There was significant heterogeneity among the studies (*I*^2^ = 75%, *P* = 0.008). The number of dissected lymph nodes was more in VATS lobectomy patients. The mean difference in the number of dissected lymph nodes was statistically significant (MD = − 6.44, 95% CI − 9.49 to − 3.40, *P* < 0.01, Fig. 10).

#### Publication bias

**Fig. 11:**
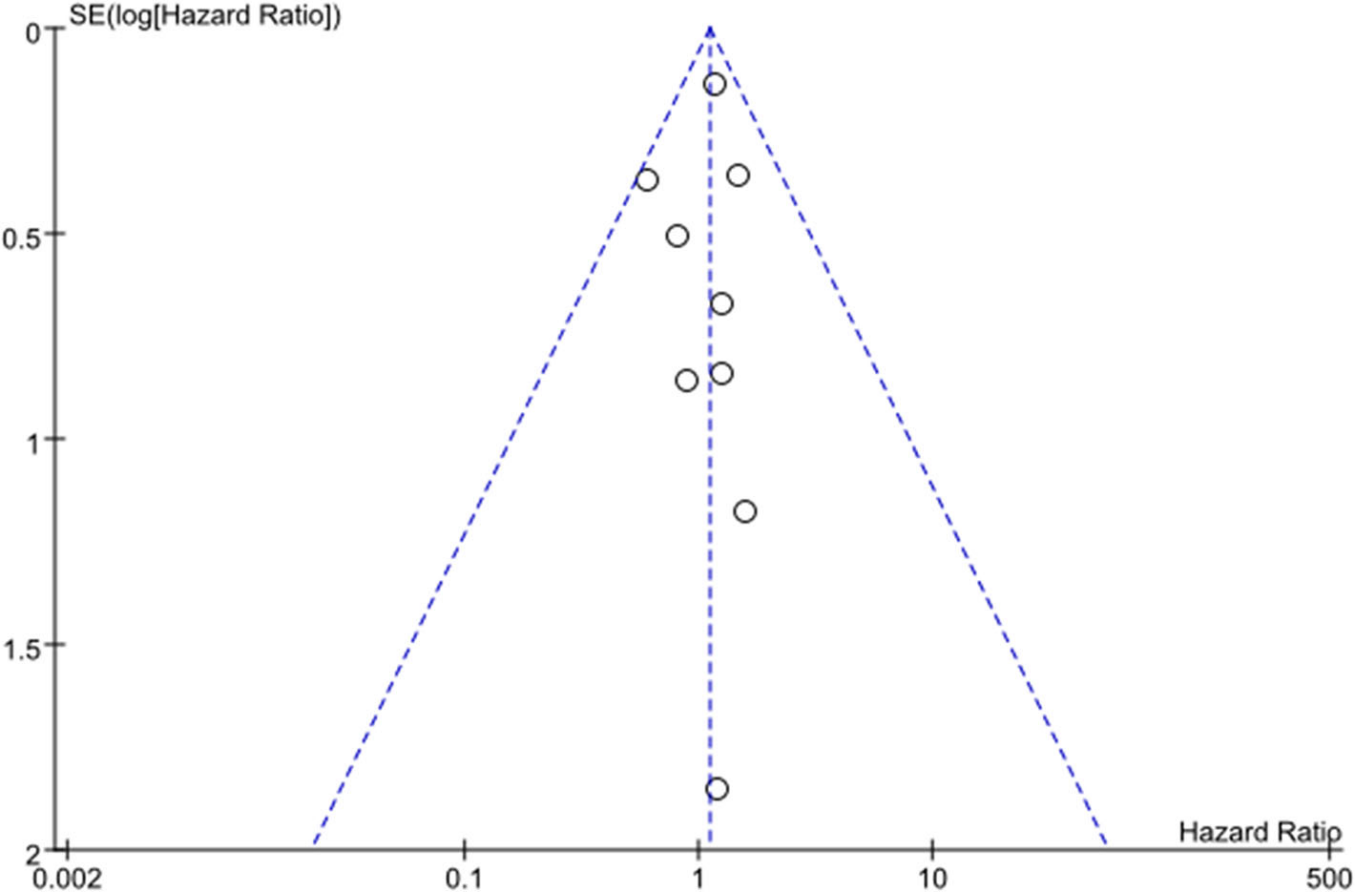
Funnel plot for overall survival of the VATS segmentectomy and VATS lobectomy groups in the studies analyzed

Funnel plots (standard error of OS) demonstrated marked symmetry, indicating absence of publication bias (Fig. 11).

## Discussion

A number of recent systematic reviews have shown that segmentectomy can achieve the same survival outcomes as lobectomy in stage I NSCLC patients [22, 23]. The study of International Early Lung Cancer Action Program [24] even suggests that the prognosis for clinical stage I tumors up to 2 cm in diameter is superior with segmentectomy. However, there is a paucity of studies on VATS segmentectomy [7, 25]. The differences between VATS segmentectomy and lobectomy in survival outcomes, postoperative complications, number of lymph nodes dissected, and so on have not been adequately studied. A recent meta-analysis [26] that compared VATS segmentectomy with VATS lobectomy included 8 articles, with a total of 463 patients who underwent VATS segmentectomy and 1150 patients who underwent VATS lobectomy. The authors found no significant difference in OS (HR = 1.03, 95% CI 0.76 to 1.39, *P* = 0.85) or DFS (HR 1.19, 95% CI 0.67 to 2.10, *P* = 0.56) between the two groups. However, the article did not analyze other outcomes.

Our meta-analysis included 12 studies that compared the perioperative and oncological outcomes of VATS segmentectomy versus VATS lobectomy in stage I NSCLC patients. Although those studies were retrospective in nature, they were all of moderate to high quality. We found that the outcomes were mostly comparable between patients undergoing VATS segmentectomy and VATS lobectomy. There were no significant differences between the two groups in survival (OS and DFS) or in perioperative outcomes such as operative time, intraoperative bleeding, air leak (> 5 days), postoperative complications, and in-hospital mortality. However, postoperative hospital stay and the number of lymph nodes dissected were both significantly lower in patients undergoing VATS segmentectomy.

The effect of segmentectomy on prognosis is debated. While segmentectomy preserves normal lung parenchyma and is therefore claimed to be beneficial for pulmonary function recovery, it is not clear whether the retained lung parenchyma serves to improve prognosis [27] or whether insufficient resection actually worsens prognosis [28]. Some studies show comparable prognosis with VATS segmentectomy and VATS lobectomy [29, 30], but others show poorer prognosis with the former [31, 32]. Most of the studies that demonstrated the superiority of lobectomy were not completely randomized and also did not consider other factors that could potentially affect survival, e.g., tumor size, type of procedure (wedge resection vs. segmentectomy), and the type of lymph node dissection. Our meta-analysis showed that retention of part of the lung parenchyma does not improve prognosis, and reduction in the extent of resection does not increase the risk of recurrence. These results are consistent with previous reports [4, 33, 34].

We found postoperative hospital stay to be significantly shorter after VATS segmentectomy. This was probably because patients who accepted VATS segmentectomy had more rapid lung recruitment and quicker return of lung function to optimal levels [35, 36]. The number of lymph nodes resected was significantly lower in VATS segmentectomy than in VATS lobectomy. This may have been because of the differences in the number of inter- and intra-segmental nodes dissected and also because lymph node sampling, rather than lymph node dissection, is generally adopted during VATS segmentectomy [20].

Most studies show that segmentectomy preserves more lung tissue and therefore promotes lung function recovery [33, 37, 38], but some reports suggest that the retained lung tissue provides little functional advantage [10, 39]. There could be several explanations. First, compensatory adaptation of the remaining lung may be better after lobectomy than after segmentectomy [40, 41]. Second, the intersegmental plane [42, 43] is created with an electrocautery device or an auto-suture device, but both methods have drawbacks, and perfect anatomical resection is not always possible; this may result in limited function of the retained lung. Unfortunately, data on lung function were not collected in this meta-analysis because there was no uniformity between the studies in the methods used for evaluation. However, this meta-analysis indicates that although retention of the lung parenchyma does not improve prognosis, it does accelerate postoperative recovery.

Some limitations of this study must be pointed out. First, all included studies were retrospective nonrandomized comparisons, with high probability of selection and reporting bias. Second, there was high heterogeneity among the studies with regard to postoperative complications, operation time, and number of dissected lymph nodes. Factors that may have been responsible for the heterogeneity include the level of experience of the surgeon and the shorter learning curve for VATS segmentectomy. The high heterogeneity can reduce the credibility of conclusions. Third, some studies did not report the precise clinical stage or explain the methods used for determining disease stage. PET-CT was used for staging of all patients in only three studies; in the other studies, some patients were staged with PET-CT and some by CT. The lack of uniformity in the methods and the uncertainty of the clinical stages might affect the reliability of our results. Fourth, some of the patients who underwent VATS segmentectomy were those who were considered unfit for lobectomy because of presence of comorbidities; this may have resulted in a selection bias and affected our results.

## Conclusion

This meta-analysis shows that VATS segmentectomy and VATS lobectomy provide similar oncological and perioperative outcomes in stage I NSCLC patients. Well-designed large randomized clinical trials, with uniform and reliable pulmonary function indicators and staging measures (e.g., PET-CT), complete complications data, and long-term postoperative follow-up, are needed to confirm the findings of this study.

## Supplementary information

**Additional file 1.** Search strategy.

**Additional file 2.** Quality assessment of all included studies.

## Data Availability

All the data used in this study can be obtained from the original articles.

## Abbreviations

DFS: Disease-free survival
HR: Hazard ratio
MD: Mean difference
NOS: Newcastle–Ottawa Scale
NSCLC: Non–small cell lung cancer
OS: Overall survival
PRISMA: Preferred Reporting Items for Systematic Reviews and Meta-Analyses
SE: Standard error
VATS: Video-assisted thoracoscopic surgery
