# Supplementary material for: Systematic review and meta-analysis of video-assisted thoracoscopic surgery segmentectomy versus lobectomy for stage I non–small cell lung cancer
Source: World J Surg Oncol. 2020 Feb 27;18:44. doi: 10.1186/s12957-020-01814-x (PMC7047378; doi:10.1186/s12957-020-01814-x)
Supplement: Supplementary file 1 — Additional file 1. Search strategy. [file 12957_2020_1814_MOESM1_ESM.docx]

**Additional file 1: Search strategy.**

**PubMed**

The database was searched on April 15, 2019, n=952

Search Strategy:

((((((((lobectomy[Title/Abstract]) AND segmentectomy[Title/Abstract]) OR lobectomy[Title/Abstract]) AND sublobar resection) OR lobectomy) AND limited resection)) AND ((((((Video-Assisted Thoracic Surgery[Title/Abstract]) OR VATS[Title/Abstract]) OR thoracoscopic[Title/Abstract]) OR thoracoscopy[Title/Abstract]) OR Thoracic Surgery Video-Assisted[Title/Abstract]))) AND ((((((((((("Lung Neoplasms"[Mesh]) OR Neoplasms, Lung[Title/Abstract]) OR pulmonary neoplasms) OR neoplasms pulmonary) OR neoplasm pulmonary) OR pulmonary neoplasm) OR lung cancer) OR cancer, lung) OR cancers, lung)))

**Web of Science**

The database was searched on April 15, 2019, n=801

Search Strategy:

1 TOPIC: （“lobectomy” AND “segmentectomy”） OR （“lobectomy” AND “sublobar resection”） OR （“lobectomy” AND “limited resection”）

2 TOPIC: “Video-Assisted Thoracic Surgery” OR “VATS” OR “thoracoscopic” OR “thoracoscopy” OR “Thoracic Surgery Video-Assisted”

3 TOPIC: “Lung Neoplasms" OR “Neoplasms, Lung” OR “pulmonary neoplasms”

OR “neoplasms pulmonary” OR “neoplasm pulmonary” OR “pulmonary neoplasm” OR “lung cancer” OR “cancer, lung”

4 #1 AND #2 AND #3

**ScienceDirect**

The database was searched on April 15, 2019, n=691.

Search Strategy:

Title, abstract, keywords: ((“lobectomy” AND “segmentectomy”） OR （“lobectomy” AND “sublobar resection”） OR （“lobectomy” AND “limited resection”）) AND (“Video-Assisted Thoracic Surgery” OR “VATS” OR “thoracoscopic” OR “thoracoscopy” OR “Thoracic Surgery Video-Assisted”) AND (“Lung Neoplasms" OR “Neoplasms, Lung” OR “pulmonary neoplasms” OR “neoplasms pulmonary” OR “neoplasm pulmonary” OR “pulmonary neoplasm” OR “lung cancer” OR “cancer, lung”)

**Cochrane Library**

The database was searched on April 25, 2019, n=45

Search Strategy:

((“lobectomy” AND “segmentectomy”） OR （“lobectomy” AND “sublobar resection”） OR （“lobectomy” AND “limited resection”）): ti,ab,kw AND (“Video-Assisted Thoracic Surgery” OR “VATS” OR “thoracoscopic” OR “thoracoscopy” OR “Thoracic Surgery Video-Assisted”): ti,ab,kw AND (“Lung Neoplasms" OR “Neoplasms, Lung” OR “pulmonary neoplasms” OR “neoplasms pulmonary” OR “neoplasm pulmonary” OR “pulmonary neoplasm” OR “lung cancer” OR “cancer, lung”): ti,ab,kw

**Scopus**

The database was searched on April 25, 2019, n=810.

Search Strategy:

TITLE-ABS-KEY : ((“lobectomy” AND “segmentectomy”） OR （“lobectomy” AND “sublobar resection”） OR （“lobectomy” AND “limited resection”）) and (“Video-Assisted Thoracic Surgery” OR “VATS” OR “thoracoscopic” OR “thoracoscopy” OR “Thoracic Surgery Video-Assisted”) and (“Lung Neoplasms" OR “Neoplasms, Lung” OR “pulmonary neoplasms” OR “neoplasms pulmonary” OR “neoplasm pulmonary” OR “pulmonary neoplasm” OR “lung cancer” OR “cancer, lung”)
