# Supplementary material for: Systematic review and meta-analysis of video-assisted thoracoscopic surgery segmentectomy versus lobectomy for stage I non–small cell lung cancer
Source: World J Surg Oncol. 2020 Feb 27;18:44. doi: 10.1186/s12957-020-01814-x (PMC7047378; doi:10.1186/s12957-020-01814-x)
Supplement: Supplementary file 2 — Additional file 2. Quality assessment of all included studies. [file 12957_2020_1814_MOESM2_ESM.docx]

**Additional file 2: Quality assessment of all included studies**

| **Study** | **Selection** | **Comparability** | **Exposure** | **Quality (score)** |
| --- | --- | --- | --- | --- |
| Hwang[10] | ★★★★ | ★★ | ★★★ | 9 |
| Echavarria[11] | ★★★ | ★★ | ★★★ | 8 |
| Landreneau[12] | ★★★ | ★★ | ★★★ | 8 |
| Nakamura[13] | ★★ | ★★ | ★★★ | 7 |
| Roman[14] | ★★★ | ★★ | ★★★ | 8 |
| Shapiro[15] | ★★★ | ★★ | ★★ | 7 |
| Song[16] | ★★★★ | ★★ | ★★★ | 9 |
| Soukiasian[17] | ★★★ | ★★ | ★★ | 7 |
| Tsubokawa[18] | ★★★ | ★★ | ★★ | 7 |
| Wang[19] | ★★★ | ★★ | ★★ | 7 |
| Yamashita[20] | ★★★★ | ★★ | ★★ | 8 |
| Zhong[21] | ★★★ | ★★ | ★★ | 7 |
